# Effects of Elevated Temperature and Carbon Dioxide on the Growth and Survival of Larvae and Juveniles of Three Species of Northwest Atlantic Bivalves

**DOI:** 10.1371/journal.pone.0026941

**Published:** 2011-10-31

**Authors:** Stephanie C. Talmage, Christopher J. Gobler

**Affiliations:** School of Marine and Atmospheric Sciences, Stony Brook University, Southampton, New York, United States of America; Smithsonian's National Zoological Park, United States of America

## Abstract

Rising CO_2_ concentrations and water temperatures this century are likely to have transformative effects on many coastal marine organisms. Here, we compared the responses of two life history stages (larval, juvenile) of three species of calcifying bivalves (*Mercenaria mercenaria*, *Crassostrea virginica*, and *Argopecten irradians*) to temperatures (24 and 28°C) and CO_2_ concentrations (∼250, 390, and 750 ppm) representative of past, present, and future summer conditions in temperate estuaries. Results demonstrated that increases in temperature and CO_2_ each significantly depressed survival, development, growth, and lipid synthesis of *M. mercenaria* and *A. irradians* larvae and that the effects were additive. Juvenile *M. mercenaria* and *A. irradians* were negatively impacted by higher temperatures while *C. virginica* juveniles were not. *C. virginica* and *A. irradians* juveniles were negatively affected by higher CO_2_ concentrations, while *M. mercenaria* was not. Larvae were substantially more vulnerable to elevated CO_2_ than juvenile stages. These findings suggest that current and future increases in temperature and CO_2_ are likely to have negative consequences for coastal bivalve populations.

## Introduction

The combustion of fossil fuels during the past two centuries has caused increases in atmospheric carbon dioxide and global temperatures, trends that are projected to continue in the coming decades [1]. Global temperatures are expected to increase 2 to 5°C this century [2]. Atmospheric CO_2_ concentrations that had increased at a rate of 1% per year in the 20^th^ century are now increasing ∼3% per year and may exceed 800 ppm by the end of this century [1], [3]. Ocean chemistry will be altered by this rising CO_2_ as levels of both pH and carbonate ions decline [4]. These changes in ocean chemistry may have transformative effects on ocean life.

Coastal zones are likely to be the first regions to experience high levels of temperature and CO_2_ predicted for the open ocean in the future due to both natural and anthropogenic processes, and some regions are already experiencing these increases. For example, upwelling can introduce water with high concentrations of CO_2_ (800–1100 ppm) along large sections of the continental shelf [5]. Acidic river water can depress carbonate ion concentrations in coastal marine environments [6]. Furthermore, many coastal regions can be net heterotrophic due to anthropogenic, terrestrial, riverine, and wetland loadings of organic carbon [7], [8], [9], [10], processes that collectively promote supersaturated CO_2_ concentrations and lower pH. Coastal water temperatures are more sensitive to extreme and rapid increases in air temperature and increases in estuarine water temperatures have outpaced those observed in the surface ocean [11], [12].

Many marine organisms, in particular those with calcified parts, can be negatively affected by acidification of ocean waters [13]. Enrichment of CO_2_ can have a negative impacts across a wide range of calcifying marine taxa from coral [14], to coccolithophores [15], echinoderms [16], and coralline algae [17]. Sediments with high levels of CO_2_ and low levels of carbonate ion have been shown to promote mortality of juvenile mollusks (*Mercenaria mercenaria* and *Mya arenaria*) [18], [19]. Elevated CO_2_ can cause decreased calcification in mussels (*Mytilus edulis*) and oysters (*Crassostrea gigas*; [20]), as well as decreased growth in mussels (*M. edulis*; [21]). Seawater enriched in CO_2_ can also depress the survival, growth, and metamorphosis of larval stages of calcifying bivalves [19], [20], [22], [23], [24], [25]. Our previous work has specifically demonstrated that larval hard clams (*M. mercenaria*) and bay scallops (*Argopecten irradians*) reared under the CO_2_ conditions representative of the pre-industrial era (250 ppm) experience significantly faster growth and metamorphosis compared to individuals exposed to modern day CO_2_ levels (390 ppm) [24], [26].

The increases in ocean temperatures projected to occur this century will impact marine life. Higher temperatures in marine ecosystems can alter primary productivity, stratification, and organismal physiology [27]. The current rate of warming in ocean waters will likely apply thermal stress to a wide range of marine organisms as the limits of their temperature tolerances are approached or exceeded [28]. Temperature is a vital factor that influences the spawning and development of invertebrate larvae and most bivalve gametes are spawned at specific temperatures [29], [30], [31]. While larval bivalves experience maximal growth and survival rates under ideal temperature conditions (e.g. ∼24°C for many northwestern Atlantic species), small increases in temperature beyond that range will depress these rates [32], [33], [34]. In addition, higher temperatures can make larval bivalves more vulnerable to other environmental stressors such as ocean acidification [35].

Concurrent, future increases in CO_2_ and water temperatures in marine environments likely may have synergistic effects on ocean life, in general, and invertebrate larvae in particular. Negative impacts of high CO_2_ are often the greatest for early life stages of many organisms, while thermal stress can affect all life stages [36]. For the tropical sea urchin, *Tripneustes gratilla*, higher temperatures increased the growth and size of larvae, while higher CO_2_ concentrations reduced calcification and negated the positive effect of higher temperatures when both temperature and CO_2_ were increased [16]. For one week old barnacles, *Semibalanus balanoides,* a significant reduction in calcification and survival was estimated under simultaneously elevated temperature and CO_2_ [37]. Red abalone larvae, *Haliotis rufescens*, displayed significant reductions in survivorship with increased CO_2_ and a brief thermal stress compared to ambient CO_2_ levels at the same thermal stress level [38]. The combination of high temperature and CO_2_ have had synergistically, negative effects on a species of arctic pteropod [39] but antagonistic impacts on crustose coralline algae [17]. Exposure of two species of oysters (*Saccostrea glomerata* and *Crassostrea gigas*) to high CO_2_ and increased temperature caused declines in fertilization success, development of embryos, and the size of larvae, as well as an increase incidence of abnormal morphology [40]. In contrast, the fertilization success of multiple species of marine invertebrates from South East Australia were unaffected by warming and ocean acidification [41]. To date, few studies have examined the simultaneous effects of CO_2_ and temperature on any species of North Atlantic marine bivalves.

Here we present experiments investigating the effects of higher seawater temperatures and past, present, and future CO_2_ concentrations on the growth and survival of the larvae of two species and juveniles of three species of CaCO_3_ synthesizing bivalves native to the east coast of North America: the hard clam or northern quahog, *Mercenaria mercenaria (Linnaeus*, 1758), the Eastern oyster, *Crassostrea virginica (Gmelin,* 1791), and the bay scallop, *Argopecten irradians (Lamarck,* 1819). These shellfish are vitally important economic resources and ecosystem engineers in shallow coastal waters [42] and performance of these early life history stages have a profound effect on the population dynamics of these animals [43], [44], [45]. Simultaneously investigating the impacts of high temperature and increasing CO_2_ concentrations permitted an evaluation of the differential vulnerability of larval and juvenile stages of each species to these environmental stressors.

## Methods

This study examined the effects of multiple CO_2_ and temperature levels on juvenile and larval stages of bivalves. For all experiments, experimental vessels with bivalves (described below) were maintained in water baths set maintained at 24 and 28°C using commercially available aquarium heaters (Aquatic Eco-systems, Inc., Florida, USA). Temperatures were recorded every 6 minutes throughout experiments using in situ data loggers (Onset©) and remained within ±0.7°C of target values. The two experimental temperatures (24 and 28°C) were chosen to represent normal and above average temperatures in Northeast US estuaries during summer months [12], [46] when larvae are spawned and juvenile stages are most likely to experience thermal stress. A gas proportionator system (Cole Parmer® Flowmeter system, multitube frame) was used to deliver CO_2_ gas to seawater treatments at multiple rates. The gas proportionator mixed appropriate flow rates of 5% CO_2_ gas, low CO_2_ gas, and pressurized air (∼390 ppm CO_2_) to yield the concentrations of carbon dioxide desired for experiments at a net flow rate that turned over experimental vessels >100 times daily. We have found that experiments performed with gases mixed via a proportionator as described here generate nearly identical seawater chemistry and larval responses compared to those obtained from tanked gases premixed at specific CO_2_ levels [26]. For experiments, the CO_2_ gas mixtures from the proportionator system were continuously delivered to the bottom of replicated (*n* = 3 or 4) experimental vessels (detailed below). With continuous bubbling, all treatment carboys remained saturated with respect to oxygen (∼8 mg L^−1^). To quantify precise CO_2_ levels attained in experimental treatments, aliquots were removed before addition of larvae as well as at the conclusion of the experiment, and analyzed during experiments using an EGM-4 Environmental Gas Analyzer® (PP Systems) system that quantified total dissolved inorganic carbon levels after separating the gas phase from seawater using a Liqui-Cel® Membrane (Membrana) a standard curve made from sodium bicarbonate. This instrument provided a methodological precision ±3.6% for replicated measurements of total dissolved inorganic carbon and provided full recovery (102±3%) of Dr. Andrew Dickson's (University of California San Diego, Scripps Institution of Oceanography) certified reference material for total inorganic carbon in seawater (Batch 102 = 2013 µmol DIC kg seawater^−1^). Levels of CO_2_ were calculated based on measured levels of total inorganic carbon, pH (mol kg seawater^−1^, NBS scale;), temperature, salinity, and first and second dissociation constants of carbonic acid in seawater according to [47] using the program CO2SYS (http://cdiac.ornl.gov/ftp/co2sys/). Daily measurements of pH (Thermo Scientific Orion Star Series™ Benchtop pH meter; ±0.002; calibrated prior to each use with NIST traceable standards; equilibrated for ∼5 minutes per sample) indicated experimental vessels maintain a constant pH level throughout experiments (<0.5% RSD within treatments). Spectrophotometric measurements of pH made using *m*-cresol purple as described by Dickson *et al.* [48] and corrected for scale [49] were never significantly different from those obtained with the high sensitivity pH microprocessor. The levels of precision for measurements of pH and DIC permitted for the accurate differentiation of CO_2_ treatment levels (see below) that differed by hundreds of ppm (250 v 390 v 750 v 1700 ppm).

### Larvae experiments

The recommendations of the ‘best practices’ for small microcosm experiments set forth by European Project on Ocean Acidification (EPOCA) were followed for this project [50]. *M. mercenaria* and *A. irradians* larvae were grown at three levels of CO_2_: a high level (∼750 ppm CO_2_), predicted for the year 2100, a modern level (∼390 ppm CO_2_), and a near pre-industrial level (∼250 ppm CO_2_), while at two different temperatures (24 and 28°C). Precise CO_2_ levels and complete carbonate chemistry from this experiment appear in Table 1. One-liter, high-density polyethylene beakers were filled with 0.2 µm filtered seawater from eastern Shinnecock Bay, New York, United States. *M. mercenaria* larvae were obtained from Cornell Cooperative Extension, Southold, NY, and *A. irradians* larvae were from the East Hampton Shellfish Hatchery, East Hampton, NY, within hours of fertilization and were distributed to each treatment beaker at a concentration of ∼350 L^−1^, consistent with post-spawning densities in estuaries (Carriker 2001). Twice weekly during experiments, larvae were gently poured onto a 64 *µ*m mesh, and the condition (live or dead) and developmental stage of each larvae (veligers, pediveligers, and metamorphosed) were determined visually under a dissecting microscope; every individual larvae was counted at every water change. Larvae from each beaker (*n* = 4, per treatment) were removed, counted, observed, and transferred into a new beaker with new filtered seawater, food, and antibiotics within a 15 minute period. Percent survivorship of all larvae was determined at each of the bi-weekly water changes when the numbers of larvae in each stage of veligers, pediveligers, and metamorphosed juveniles were quantified. Dead larvae were characterized by a lack of swimming and movement of the velum and, when visible, internal organs, as well as a loss of pigmentation and fully open valves. Experiments were terminated after at least 50% of the surviving larvae in all treatments had metamorphosed, which averaged three weeks among all experiments. To determine the percentage of individuals that had metamorphosed at each time point, the following equation was employed:
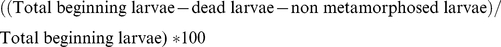



**Table 1:** Mean temperature, pH, carbonate chemistry, alkalinity, and salinity (±1 SD) during the three-level carbon dioxide and two-level temperature experiments with *Mercenaria mercenaria,* and *Argopecten irradians* larvae.

Parameter	Pre-industrial CO _2_	Ambient, present day CO_2_	Elevated CO_2_
*Mercenaria mercenaria*			
Temperature (°C)	24±0.7	24±0.7	24±0.7
pH	8.210±0.032	8.081±0.042	7.8±0.012
pCO_2_ (ppm)*	220.4±24.235	375.3±36.45	771.6±29.113
Ω_calcite_ *	2.86±0.50	2.68±0.51	1.51±0.15
Ω_aragonite_ *	1.84±0.37	1.72±0.36	0.98±0.13
Total DIC (*µ*mol L^1^)	1115.3±95.67	1374.1±62.89	1439.4±31.38
CO_3_^2−^ (*µ*mol L^−1^)*	112.7±21.24	105.5±26.23	59.7±9.806
Alkalinity (TA) (*µ*mol kg^1^)*	1296.8±121.3	1527.3±86.56	1509.3±27.69
Salinity	28.0±1.0	28.0±1.0	28.0±1.0
*Argopecten irradians*			
Temperature (°C)	24±0.7	24±0.7	24±0.7
pH	8.200±0.026	8.080±0.059	7.810±0.016
pCO_2_ (ppm)*	238.4±25.012	373.9±41.540	756.2±19.986
Ω_calcite_ *	2.95±0.16	2.66±0.57	1.55±0.12
Ω_aragonite_ *	1.9±0.42	1.72±0.45	1.00±0.24
Total DIC (umol L^−1^)	1176±56.27	1368.7±36.99	1517±35.45
CO_3_^2−^ (*µ*mol L^1^)*	133.7±22.32	105.1±28.52	61.3±12.321
Alkalinity (TA) (*µ*mol kg^1^)*	1359.6±35.98	1521.4±55.06	1517.1±46.66
Salinity	28.0±1.0	28.0±1.0	28.0±1.0
*Mercenaria mercenaria*			
Temperature (°C)	28±0.7	28±0.7	28±0.7
pH	8.200±0.040	8.090±0.046	7.8±0.012
pCO_2_ (ppm)*	247.4±16.241	379.0±43.12	794.6±29.113
Ω_calcite_ *	3.43±0.53	3.17±0.56	1.75±0.15
Ω_aragonite_ *	2.24±0.99	2.07±0.45	1.14±0.13
Total DIC (*µ*mol L^1^)	1196±76.24	1389.2±53.45	1439.6±31.38
CO_3_^2−^ (*µ*mol L^−1^)*	133.7±20.34	123.4±36.42	68.1±9.806
Alkalinity (TA) (*µ*mol kg^1^)*	1404.3±123.61	1568.2±66.49	1522.8±27.69
Salinity	28.0±1.0	28.0±1.0	28.0±1.0
*Argopecten irradians*			
Temperature (°C)	28±0.7	28±0.7	28±0.7
pH	8.210±0.029	8.08±0.054	7.810±0.026
pCO_2_ (ppm)*	239.8±13.078	386.7±44.23	772.7±29.951
Ω_calcite_ *	3.48±0.17	3.09±0.57	1.78±0.16
Ω_aragonite_ *	2.27±0.78	2.01±0.42	1.16±0.14
Total DIC (umol L^−1^)	1189.1±53.57	1557.1±32.88	1433.6±30.21
CO_3_^2−^ (*µ*mol L^1^)*	135.7±43.2	120.3±28.46	69.3±12.321
Alkalinity (TA)(*µ*mol kg^1^)*	1400.8±65.25	1557.1±70.21	1519.4±36.45
Salinity	28.0±1.0	28.0±1.0	28.0±1.0

*Parameters calculated using CO2SYS.

Larvae were fed an ideal food source at a density known to maximize bivalve larval growth and survivorship through metamorphosis [24], [34], [51]. Cultures of *Isochrysis galbana* (Tahitian strain, T-Iso) were maintained in exponential phase growth using standard culture conditions and added at a density of 2×10^4^ mL^−1^ daily to each experimental beaker as a food source. To promote high survivorship, all containers in contact with larvae were never exposed to chemicals or detergents [24]. To discourage the growth of bacteria during experiments, an antibiotic solution (Sigma-Aldrich No. 4083, 5000 units of Penicillin, 5 mg of Streptomycin, and 10 mg of Neomycin per milliliter of solution) was added to each beaker at 1% its original concentration at the beginning of each experiment and at the time of each water change (approximately 2 times weekly). This antibiotic mixture at this concentration has been shown to have no negative effects on the growth and survivorship of shellfish larvae [24]. Experiments presented here were repeated without antibiotic treatments and yielded no difference in bivalve larval survival suggesting that neither the antibiotics nor the bacteria in seawater altered the results presented here. To meet the assumption of normality and homogeneity, survival and percent metamorphosed data were arc-sin square root transformed after which a two-way ANOVAs was performed where temperature and CO_2_ were the main effects. Sizes of larvae were also examined via two-way ANOVAs. Post-hoc Tukey multiple comparison tests were performed to examine the differences among percent survival, percent metamorphosis, and sizes at each temperature and CO_2_ level. Statistical analyses were performed with SYSTAT 13 © Copyright, 2009, Systat Software, Inc.

To estimate the relative lipid content of larvae, Nile Red dye was used to bind to neutral lipids and fluoresce under an FITC filter on an epifluorescent microscope [51], [52]. A Nile Red stock solution was made of 1.25 mg of Nile Red crystals in 100 ml of acetone. Randomly selected larvae (*n* = 15) from each replicated treatment bottle (*n* = 12) were stained with a 1∶9 dilution of the stock solution and 0.2 µm filtered seawater. Larvae were exposed to the stain for ∼1.5 hours during which larval motion ceased, permitting the uniform, planar orientation of each individual for image analyses. Larvae were digitally photographed with a Roper Scientific Photometrics CoolSNAP ES camera mounted to an epiflorescent microscope. Digital images of each larva were analyzed for the area of lipid accumulation and the diameter and the area of individuals using Image J® software. Diameters were measured on randomly selected larvae (*n* = 15) from each replicated treatment vessel (*n* = 12). A lipid index was estimated by dividing the area of the larvae containing the fluorescing lipids by the total larval area thereby allowing for direct comparisons among treatments. Two-way ANOVAs and post-hoc Tukey multiple comparison tests were performed to examine the differences among larval lipid indexes, as well as shell length at each CO_2_ level.

### Juvenile experiments

Juvenile bivalves were obtained during early summer from the East Hampton Shellfish Hatchery, East Hampton, NY. Starting mean lengths and ash-free, dry weights (± standard deviation) of individuals were 6.09±0.65 mm and 1.36±0.048 g for *M. mercenaria*, 11.48±3.60 mm and 1.48±0.221 g for *C. virginica*, and 15.93±1.59 mm and 1.74±0.172 g for *A. irradians*. Ten individuals of each species were placed into triplicate, 10-liter, high-density polyethylene vessels that were maintained in water baths of 24 or 28°C (Table 2). CO_2_ was continuously delivered as described above at ∼400 and 1700 ppm representing ambient, pelagic CO_2_ found today and a high concentration that our atmosphere may approach in the future [53], but within the range of levels found in and near the benthos which is frequently undersaturated with regarding to carbonate [6], [18], [19]. The range of CO_2_ used in this experiment (∼400–1700 ppm) is also commonly found in nearshore and estuarine marine environments [7], [8], [9], [10]. Experimental vessels were bubbled with appropriate CO_2_ levels for 24 h prior to commencing experiments. Precise CO_2_ levels and complete carbonate chemistry from this experiment appear in Table 2. Each juvenile introduced into each treatment was identified with colored paint, allowing growth of individuals to be assessed through the 45 day experiment, a duration matching peak, hot, summer temperature in temperate estuaries [12], [46]. Every three days, water was exchanged with ambient sea water from Old Fort Pond, Southampton, NY, USA, or Northwest Harbor in East Hampton, NY, USA (salinities = 28±3). Newly collected water was bubbled for 12 h prior to transferring individual bivalves to new vessels. Nutrients (10 µM nitrate and 0.63 µM orthophosphate) were added immediately and daily to experimental vessels that were held under a bank of fluorescent lights that were on an ∼12∶12 h light∶dark cycle and delivered a light intensity of ∼10 µmol quanta m^−2^ s^−1^ to encourage phytoplankton growth. Consequently, chlorophyll *a* measured using standard methods at the start and end of each water change during experiments [54] averaged 9.8±3.7 µg L^−1^ and never fell below 5 µg L^−1^, a level generally deemed adequate for maximal growth rate of juvenile bivalves [55], [56], [57].

**Table 2:** Mean temperature, pH, carbonate chemistry, alkalinity, and salinity (±1 SD) during the two-level carbon dioxide and two-level temperature experiments with *Mercenaria mercenaria, Crassostrea virginica,* and *Argopecten irradians* juveniles.

Parameter	Ambient, present day CO_2_	Elevated CO_2_
*Mercenaria mercenaria, Crassostrea virginica,* and *Argopecten irradians* juveniles		
Temperature (°C)	24±0.65	24±0.65
pH	8.091±0.001	7.620±0.060
pCO_2_ (ppm)*	400±12.34	1665±25.60
Ω_calcite_ *	2.99±0.10	1.42±0.18
Ω_aragonite_ *	1.93±0.07	0.92±0.12
Total DIC (*µ*mol L^1^)	1502±48.47	2023±29.01
CO_3_^2−^ (*µ*mol L^−1^)*	117.9±3.95	56.1±7.16
Alkalinity (TA)(*µ*mol kg^1^)*	1667.5±52.15	2052.2±19.39
Salinity	28.0±3.0	28.0±3.0
Temperature (°C)	28±0.65	28±0.65
pH	8.092±0.002	7.617±0.047
pCO_2_ (ppm)*	399.5±1.68	1737±18.71
Ω_calcite_ *	3.38±0.04	1.64±0.19
Ω_aragonite_ *	2.20±0.03	1.07±0.12
Total DIC (*µ*mol L^1^)	1473±11.61	2039±4.10
CO_3_^2−^ (*µ*mol L^−1^)*	131.5±1.64	64.0±7.16
Alkalinity (TA) (*µ*mol kg^1^)*	1659.4±13.52	2080.8±17.67
Salinity	28.0±3.0	28.0±3.0

*Parameters calculated using CO2SYS.

Tissue and shell weight of juvenile bivalves was quantified by drying individuals for 72 hours at 60°C followed by combustion for 4 hours at 450°C. Individuals that did not survive the duration of the experiment were removed immediately, frozen, and then weighed with individuals surviving the duration of the experiment. The post-combustion weight represented the shell weight whereas the difference between the dry and combusted weights represented organic tissue weight. Tissue and shell weight-based growth rates were calculated by dividing the change in weight by the duration of the experiment in days. Growth rates were compared by means of two-way ANOVAs where temperature and CO_2_ were the main effects. Post-hoc Tukey multiple comparison tests were performed to examine the differences among juvenile growth at each temperature and CO_2_ level. Survival of individuals was assessed daily and dead individuals (*A. irradians* only during the final weeks of the experiment) were removed in <24 hr of expiring. The percent mortality of *A. irradians* within each treatment was arc-sin square root transformed after which a two-way ANOVA was performed where temperature and CO_2_ were the main effects.

## Results

**Figure 1:**
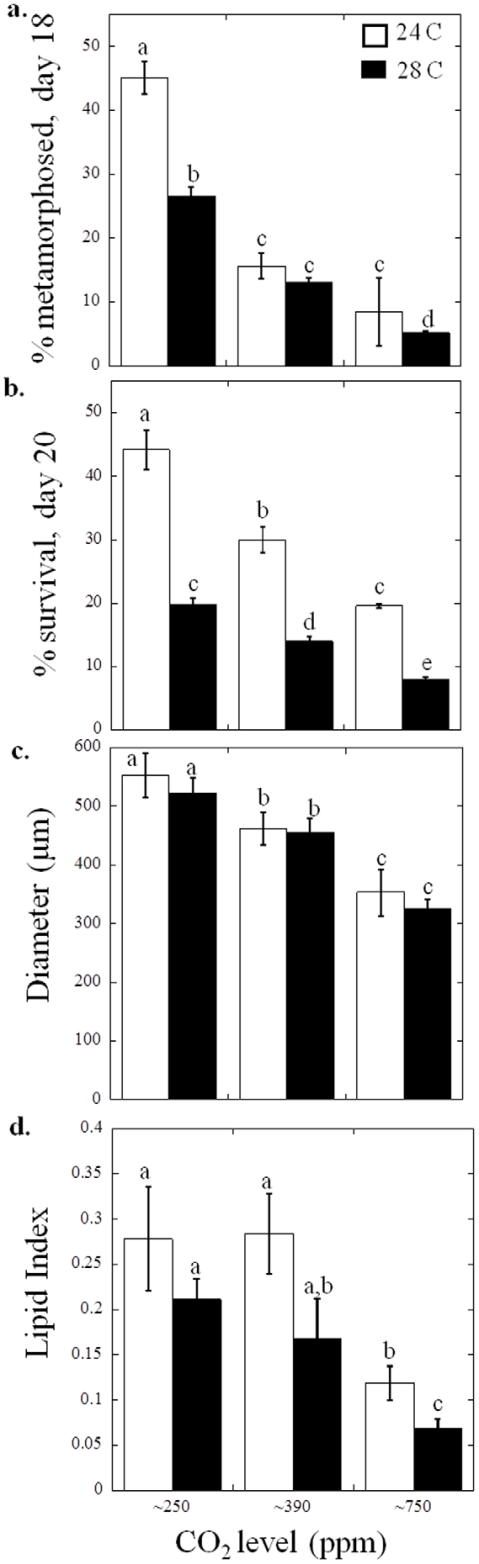
Performance of *Mercenaria mercenaria* larvae grown under three levels of CO_2_, approximately 250, 390, and 750 ppm, and two temperatures 24°C (white bars) and 28°C (black bars; see **Table 1** for carbonate chemistry). a. Percent metamorphosed of individuals 18 days post- fertilization, b. Percent larval survival (20 days post-fertilization), c. Diameters of larvae (20 days post-fertilization), and d. Lipid index (lipid area/total area) (20 days post-fertilization). Error bars represent standard deviation of replicated vessels per treatment (*n* = 4 per treatment), and for Tukey multiple comparisons, *p*≤0.05. Statistical results were based on arcsine square root transformations of the % data for a. and b.

Carbon dioxide and temperature both significantly affected larval metamorphosis (*p*<0.001; two-way ANOVA, Table S1), survival (*p*<0.001; two-way ANOVA, Table S1), growth (*p*<0.001; two-way ANOVA, Table S1) and lipid synthesis (*p*<0.001; two-way ANOVA, Table S1). In *M. mercenaria* larvae, temperature and CO_2_ had a significant, slightly antagonistic, interactive effect on *M. mercenaria* metamorphosis (*p*<0.001; two-way ANOVA, Table S1). The percentage of individuals that had metamorphosed and survived, as well as individual grow rates were all highest for individuals grown under 250 ppm and at 24°C and were lowest for individuals grown at 750 ppm CO_2_ and 28°C (Fig. 1, S1). For example, 18 days post-fertilization, 45±2.6, 16±2.0, and 8±5.3% of individuals (± standard deviation) at 24°C had metamorphosed under ∼250, 390, 750 ppm CO_2_, where as 27±0.6, 13±0.4, and 5±0.3% had done so at ∼250, 390, 750 ppm CO_2_ and 28°C (Fig. 1a, S1). With increasing CO_2_ values (∼250, ∼390, and ∼750 ppm), larval survival decreased from 44±3.1 to 30±2.1 and 20±0.3% at 24°C compared to 20±0.9 to 14±0.5 and 8±1.5% at 28°C (*p*<0.05 for all, Fig. 1b.). For *M. mercenaria* larvae, there was a synergistic interaction (*p*<0.001; two-way ANOVA, Table S1) between CO_2_ and temperature, as survival percentages in this combined treatment were lower than expected from the individual treatments. Regarding size, *M. mercenaria* larvae at 24°C and ∼250 ppm CO_2_ had mean diameters of 553±38 µm while increasing temperatures and CO_2_ level progressively depressed sizes with individuals grown at 28°C and ∼750 ppm CO_2_ having mean diameters of 325±22 *µ*m (Fig. 1c). Lipid indices for *M. mercenaria* were always higher at 24°C (0.23±0.09) compared to larvae grown at 28°C (0.15±0.07; *p*<0.001, two-way ANOVA; Table S1, Fig. 1d). The lipid content for *M. mercenaria* larvae also decreased with increasing CO_2_ levels (*p*<0.001, two-way ANOVA; Table S1, Fig. 1d). While there was no significant differences in lipid indices between ∼250 and ∼390 ppm at either temperature, there was a significant decrease in lipid indices when the CO_2_ level was enriched from ∼250 or ∼390 to ∼750 ppm for both temperatures (Fig. 1d).

**Figure 2:**
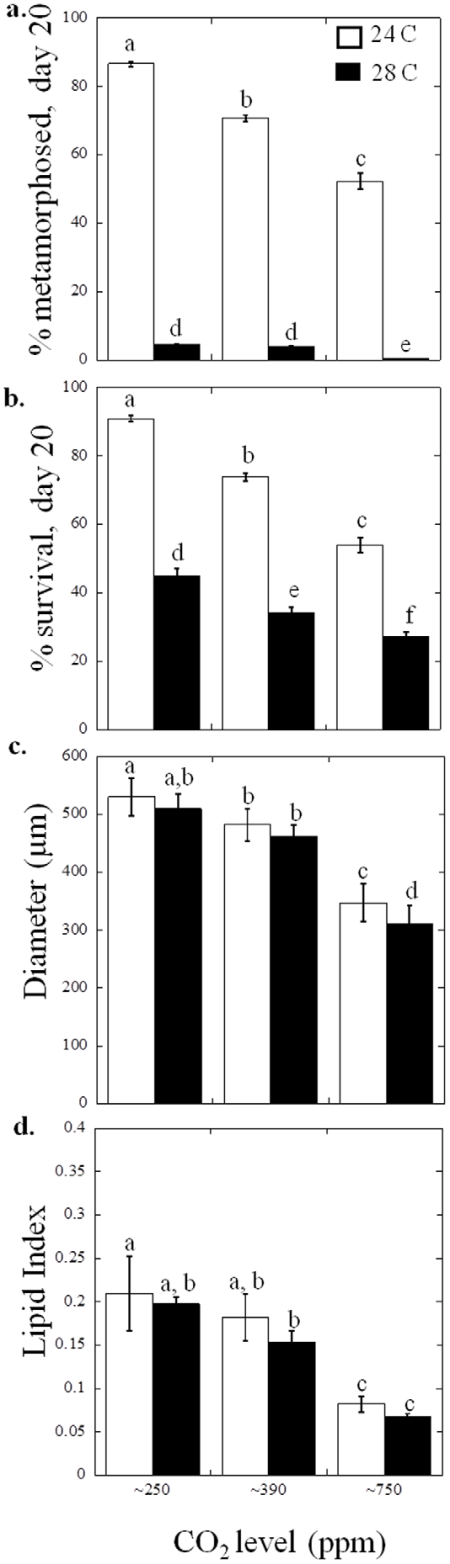
Performance of *Argopecten irradians* larvae grown under three levels of CO_2_, approximately 250, 390, and 750 ppm, and two temperatures 24°C (white bars) and 28°C (black bars; see **Table 1** for carbonate chemistry). a. Percent metamorphosed of individuals 18 days post- fertilization, b. Percent larval survival (20 days post-fertilization), c. Diameters of larvae (20 days post-fertilization), and d. Lipid index (lipid area/total area) (20 days post-fertilization). Error bars represent standard deviation of replicated vessels per treatment (*n* = 4 per treatment), and for Tukey multiple comparisons, *p*≤0.05. Statistical results were based on arcsine square root transformations of the % data for a. and b.

Responses of *A. irradians* larvae to temperature and CO_2_ levels were similar to *M. mercenaria* and in some cases were more dramatic. There was a significant decrease in the percent of individual *A. irradians* larvae that had developed into metamorphosed juveniles with increasing CO_2_ and increasing temperature (*p*<0.001; two-way ANOVA, Table S1), as well as a synergistic interaction between both temperature and CO_2_ concentrations for larval metamorphosis (*p*<0.001; two-way ANOVA, Table S1). While 87±0.8 and 71±0.9 and 53±2.3% individuals had metamorphosed after 20 days at 24°C and ∼250, ∼390, and ∼750 ppm, respectively, fewer than 10% of individuals did so at 28°C with fewer than 0.5% metamorphosed at 28°C and ∼750 ppm CO_2_ (Fig. 2a, S2). There was also a significant decline in larval survival with each increased CO_2_ and temperature level (*p*<0.001; two-way ANOVA; Table S1, Fig. 2b). There was also a slightly antagonistic interactive effect of CO_2_ and temperature on the percentage of *A. irradians* larval survival (*p*<0.001; two-way ANOVA, Table S1). At 24°C, 91±0.9, 74±1.1, and 54±2.3% of individuals survived at 250, ∼390, and ∼750 ppm, respectively, whereas at 28°C, 45±0.8, 35±0.4, and 27±0.9±% of individuals survived, respectively (Fig. 2b). Higher CO_2_ and temperature depressed the size attained by *A. irradians* larvae (*p*<0.001; two-way ANOVA, Table S1). Mean diameters of *A. irradians* larvae at 24°C and ∼250 ppm were 530±33 *µ*m while sizes progressively decreased with higher temperature and CO_2_ levels to 309±33 µm at 28°C and ∼750 ppm (*p*<0.05, Tukey for all; Fig. 2c). For *A. irradians* larvae, there were significant differences in lipid indices among CO_2_ levels (*p*<0.001, two-way ANOVA, Table S1), and between the two temperatures (*p*<0.05, two-way ANOVA, Table S1). At both temperatures, lipid indices in *A. irradians* larvae decreased from 0.21±0.04 to 0.18±0.03 to 0.08±0.01 as CO_2_ levels increased from ∼250 to ∼390 and ∼750 ppm (*p*<0.001 for ∼250 or ∼390 compared to ∼750 ppm CO_2_; Fig. 2d). At 28°C, lipid indices decreased from 0.19±0.007 to 0.15±0.01 to 0.07±0.004 as CO_2_ levels increased from ∼250 to ∼750 pm (Fig. 2d).

**Figure 3:**
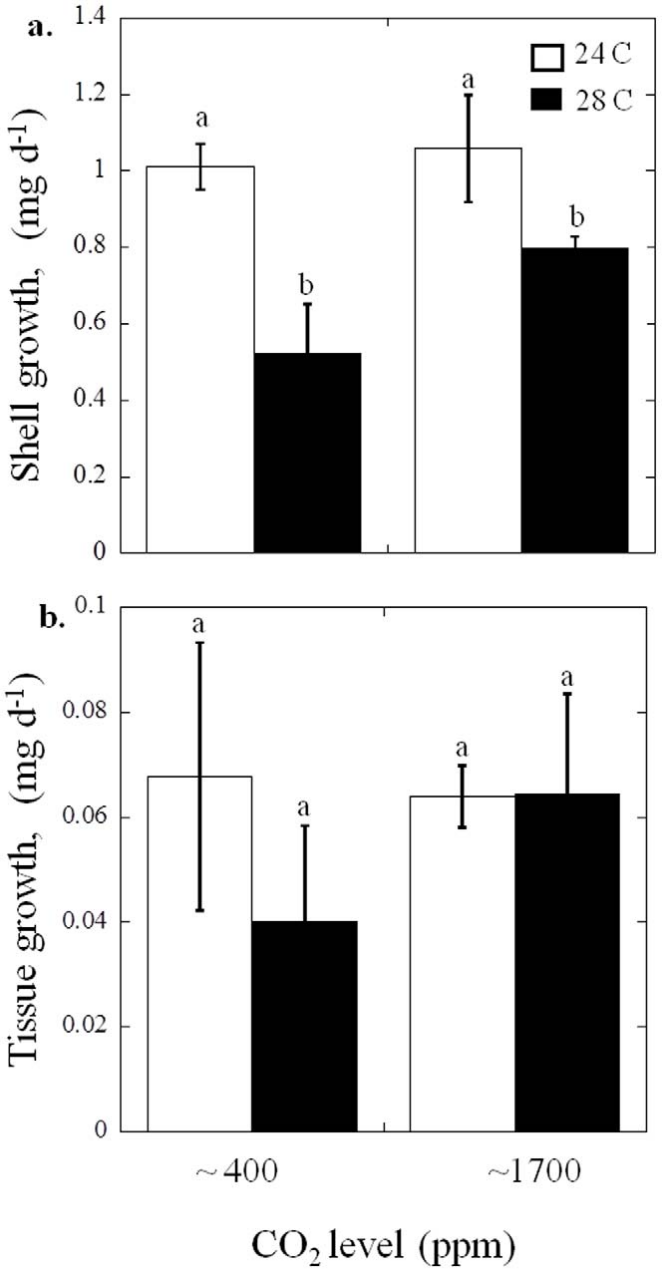
Growth of *Mercenaria mercenaria* juveniles at two levels of CO_2_, approximately 400 and 1700 ppm, and two temperatures 24°C (white bars) and 28°C (black bars; see **Table 2** for carbonate chemistry). a. Shell growth and b. Tissue growth. Error bars represent standard deviation of replicated vessels per treatment (*n* = 3 per treatment).

Unlike the larvae, juvenile *M. mercenaria* were unaffected by even higher levels of CO_2_, but were affected by temperature differences. For example, the shell growth of juvenile *M. mercenaria* was significantly greater at 24°C (1.03±0.06 mg d^−1^) compared to 28°C (*p*<0.01, two-way ANOVA; Table S1, Fig. 3a). Tissue growth for *M. mercenaria* juveniles was not significantly altered by temperature and CO_2_ did not significant alter shell or tissue growth of *M. mercenaria* juveniles (Table S1, Fig. 3b).

**Figure 4:**
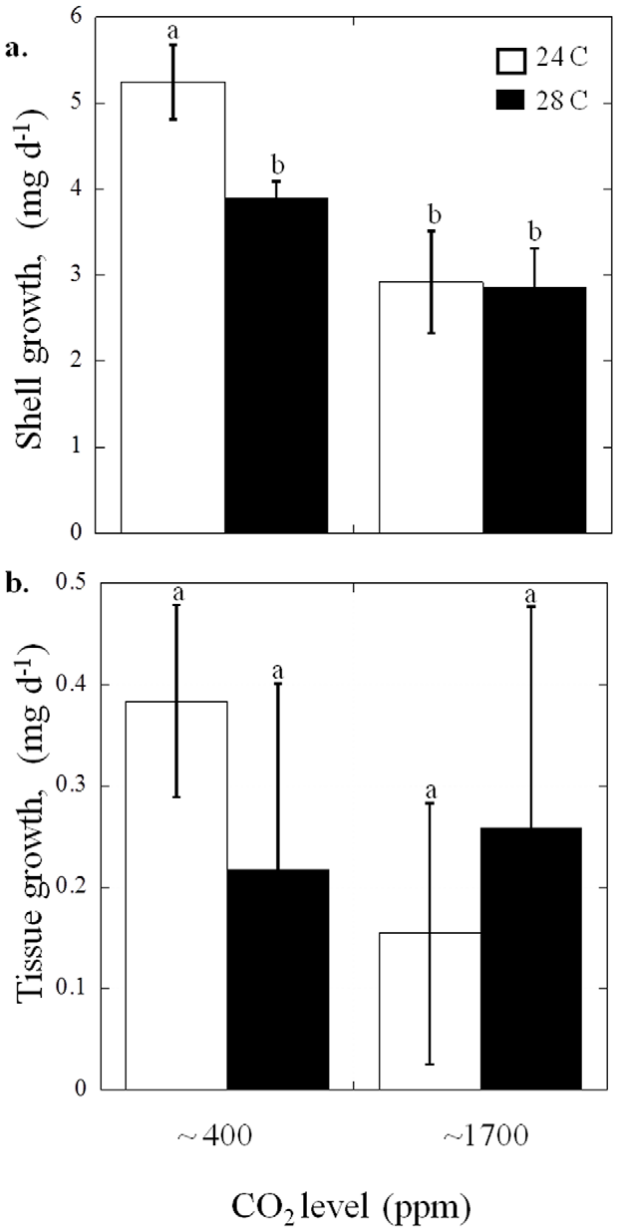
Growth of *Crassostrea virginica* juveniles at two levels of CO_2_, approximately 400 and 1700 ppm, and two temperatures 24°C (white bars) and 28°C (black bars; see **Table 2** for carbonate chemistry). a. Shell growth and b. Tissue growth. Error bars represent standard deviation of replicated vessels per treatment (*n* = 3 per treatment).

Unlike *M. mercenaria*, shell growth of *C. virginica* juveniles was significantly lower at 1700 ppm CO_2_ (2.88±0.10 mg d^−1^) compared to 400 ppm CO_2_ (4.57±0.17 mg d^−1^; *p*<0.05; two-way ANOVA; Table S1, Fig. 4a). Tissue growth for *C. virginica* juveniles was not significantly affected by temperature or CO_2_ (Table S1, Fig. 4b).

**Figure 5:**
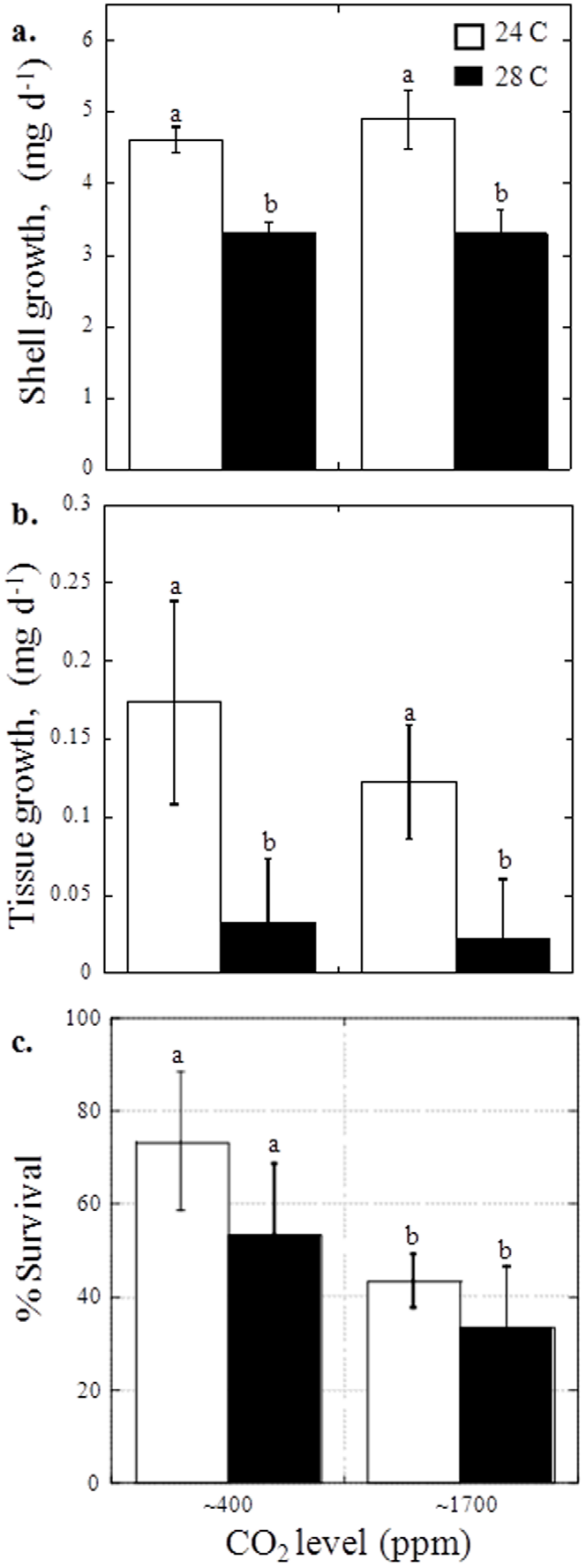
Growth and survival of *Argopecten irradians* juveniles at two levels of CO_2_, approximately 400 and 1700 ppm, and two temperatures 24°C (white bars) and 28°C (black bars; see **Table 2** for carbonate chemistry). a. Shell growth, and b. Tissue growth, c. Percent survival of individuals after 45 days. Error bars represent standard deviation of replicated vessels per treatment (*n* = 3 per treatment).

Juvenile *A. irradians* were sensitive to both elevated CO_2_, and elevated temperatures treatments used in this study. With increasing temperature from 24 to 28°C, *A. irradians* juvenile shell growth decreased from 4.75±0.17 mg d^−1^ to 3.30±0.13 mg d^−1^ while tissue growth decreased from 0.14±0.02 mg d^−1^ to 0.03±0.002 mg d^−1^ (*p*<0.05; two-way ANOVA; Table S1, Fig. 5a). Although CO_2_ did not significantly alter shell- or tissue-based growth in juvenile *A. irradians* (Table S1), the higher CO_2_ and temperature yielded a significant interactive, decline in juvenile *A. irradians* survival from 73.3±15% and 53.3±15.3% for 24 and 28°C, respectively, at 400 ppm CO_2_, to 43.3±5.8% and 33.3±13.0% for 24 and 28°C, respectively, at 1700 ppm (*p*<0.05; two-way ANOVA for CO_2_ only, Table S1, Fig. 5c). Survival of juvenile *M. mercenaria* and *C. virginica* juveniles was very high (97±6% and 93±6%, respectively) and was not significantly altered by temperature or CO_2_ (Table S1).

## Discussion

Global climate change has acidified and warmed the oceans, trends that are projected to continue this century. Anthropogenic processes, proximity to terrestrial carbon sources, and the shallow nature of coastal ecosystems make them currently vulnerable to temperatures and CO_2_ increases that may not occur in open ocean waters for many decades. This study demonstrates, for the first time, that elevated levels of CO_2_ and temperature negatively impact both juvenile and larval stages of bivalves. Larvae were found to be more sensitive to elevated levels of CO_2_ and temperature than juvenile stages and unlike two of the three juvenile species investigated, the effects of CO_2_ and temperature were additive for larvae. The high temperature (28°C) and high CO_2_ (∼750 ppm) treatment yielded the lowest survival, growth, metamorphosis, and lipid accumulation for both larval species. Collectively, these results provide novel insight regarding the effects of CO_2_ and temperature on the survival and development of multiple bivalves in coastal ecosystems.

Larvae represent a critical life stage for shellfish populations as reductions in the growth and survival of larvae have the potential to translate into substantial declines in adult populations [44], [45], [58], [59]. Temperature has a primary influence on the spawning, growth, and development of bivalve larvae. *M. mercenaria* adults from New York to Connecticut waters are known to spawn when summer water temperatures reach 23–25°C [30] while in temperate populations of *A. irradians* spawning is typically triggered by water temperatures close to 23°C [29], [60]. Resultant larvae grow optimally at temperatures around 24–25°C, but may experience slowed growth and even enhanced mortality at higher temperatures [32]. Consistent with this finding, *M. mercenaria* and *A. irradians* larvae experienced significant declines in survival, growth, and metamorphosis at 28°C compared to 24°C during this study. In the future, hotter summer water temperatures (I.P.C.C. 2007; Fussel 2009) may present bivalves with a smaller window of opportunity for optimal larval growth.

Prior studies have demonstrated that increases in CO_2_ concentrations beyond levels found in today's surface oceans have negative impacts on juvenile [18], [20] and larval bivalves [22], [24], [26], [61]. The present study revealed similar trends and confirmed our prior finding that pre-industrial CO_2_ levels provide maximal performance in larval hard clams and bay scallops [24], [26] as there were declines in survival, metamorphosis, diameter, and lipid indices for both *M. mercenaria* and *A. irradians* larvae at CO_2_ concentrations above ∼250 ppm (Fig. 1 and 2). Exposure of shellfish larvae to higher temperatures can make them more vulnerable to other stressors such as pollutants [35] and consistent with this, the simultaneous increase in temperature and CO_2_ depressed survival, metamorphosis, growth and lipid content of larvae beyond the effect of either individual treatment (Fig. 1 and 2). This was most dramatically represented by *A. irradians* larvae that displayed only 10% mortality under 24°C and 250 ppm CO_2_ compared to >70% morality of individuals exposed to 28°C and ∼750 ppm CO_2_ (Fig. 2). *A. irradians* populations are known to display boom and bust cycles that have been previously attributed to disease [62], overfishing [63], and/or harmful algae [64]. Our results demonstrate that interannual variability in temperature and CO_2_ are also likely to promote such cycles. Within an ecosystem setting the net effects of higher temperature and CO_2_ on bivalve larval survival may be more profound than measured during our experiments since larvae with extended metamorphosis times, that are smaller, and/or that accumulate fewer lipids, all symptoms of larvae reared at high temperature and CO_2_, are more likely to perish once settled [34], [52], [65], [66]. In the past decade, temperate coastal waters have experienced periods of high temperatures (three weeks >28°C in NY in 2010; C. Flagg, Stony Brook University, unpublished data) that match the duration of typical bivalve larval development periods [30], [34]. Since such high temperatures can be coupled with CO_2_ levels exceeding 1,000 ppm [5], [24] the negative effects of high temperature and high CO_2_ may already be impacting coastal marine bivalve populations [26].

Adult populations of the three bivalve species examined in this study exist over a wide range of temperatures. *C. virginica* adults are tolerant of temperatures from −2 to 36°C and the geographical distribution of this species extends from the Gulf of St. Lawrence to the Gulf of Mexico [67] with growth being most rapid in the warmer waters found at its southern extent [68]. *M. mercenaria* distributions extend from the Gulf of St. Lawrence south to the Florida Keys, and this species can survive from 0 to 30°C [69]. Water temperatures between 20 and 24°C, however, have proven to provide maximal growth rates for *M. mercenaria* [70], [71] with levels above 24°C yielding reduced pumping rates [72] and depressed growth rates of juvenile populations [57]. *A. irradians* and populations of *A. irradians* subspecies can be found from Cape Cod, Massachusetts into the Gulf of Mexico [73] and prolonged exposure of all life stages to 30°C can promote mortality in this species [74], [75]. With global warming, shallow water habitats are experiencing extended periods of high temperature that heightens physiological stress for bivalves [76]. During this study, temperatures of 28°C decreased the growth and survival of juvenile *M. mercenaria* and *A. irradians,* respectively (Fig. 3 and 5), a temperature known to be detrimental to juvenile stages of these species [72], [73]. In contrast, juvenile *C. virginica* growth was not reduced at 28°C (Fig. 4), a finding consistent with this species' ability to thrive in warmer waters [68].

Some of the differential susceptibility to high CO_2_ among bivalve species seemed consistent with their position in the benthos. *M. mercenaria* juveniles and adults are infaunal being commonly burrowed in sediments that can be undersaturated with respect to carbonate [18]. Consistent with being well-adapted to such exposure, juvenile *M. mercenaria* growth was unaffected by the high levels of CO_2_ administered during our experiment, despite aragonite being slightly undersaturated during the experiment (Ω = 0.92). In contrast, high CO_2_ significantly depressed the growth and survival of juvenile *C. virginica* and *A. irradians*, respectively (Figs. 4 and 5), two epifaunal species that are less likely to encounter sediments undersaturated with respect to calcium carbonate compared to infaunal species. Recent studies indicate many epifaunal species will encounter or already have encountered environments undersatruated with respect to calcium carbonate that may already be altering bivalve population structure [6], [77], [78]. In addition, two other epifaunal species (blue mussels, *Mytilus edulis*, and the Pacific oyster, *Crassostrea gigas*) have experienced decreased calcification and survival under high CO_2_ concentrations [20], [21]. Similar to these epifaunal bivalves, *C. virginica* may also be calcifying less under increased concentrations of CO_2_ leading to the depressed growth rates observed during this study.

With regard to temperature, it may be hypothesized that epifaunal species are less sensitive to high temperatures since they are commonly exposed to warmer temperatures within shallow estuaries whereas infaunal species may avoid high temperatures by burrowing into cooler in sediment [79]. This could partly account for the significant decline in growth for the normally infaunal *M. mercenaria* at higher temperatures but an absence of a temperature affect on the epibenthic eastern oysters, *C. virginica*. We observed a different trend, however, for juvenile *A. irradians*, which were highly sensitive to prolonged exposure to both high temperature and high CO_2_. Consistent with this finding, the early development the epifaunal oyster, *Saccostrea glomerata*, was negatively affected by both high temperature and high CO_2_ while *Crassostrea gigas* was more resistant to these stressors [40], [80]. Furthermore, a study of *C. virginica* juveniles native to Chesapeake Bay reported that higher temperatures and CO_2_ additively decreased calcification rates [78]. Therefore, it would seem factors beyond life history-facilitated adaptations influence the vulnerability of bivalves to high temperature with and without high CO_2_ and that mollusks are differentially adapted to these environmental stressors.

For *M. mercenaria* and *A. irradians*, the responses of the larval and juvenile stages to increased temperature and increased CO_2_ concentrations may be compared. For *M. mercenaria* larvae, survival declined by 82% as conditions changed from low temperature and CO_2_ to 28°C and 750 ppm CO_2_. In contrast, survival juvenile *M. mercenaria* was unaffected by 28°C and even higher levels of CO_2_ (∼1700 ppm). *A. irradians* larval survival declined by 70% as conditions changed from low temperature and CO_2_ to 28°C and 750 ppm CO_2_ while juvenile *A. irradians* displayed a 50% reduction in survival when 24°C, ∼400 ppm CO_2_ treatments were compared to 28°C, and ∼1700 ppm CO_2_ treatments, a level more than two-fold higher than the concentration larvae were exposed to. Therefore, the larval stages of both species were substantially more sensitive to high temperature and CO_2_ than juvenile stages. The greater sensitivity of bivalve larvae compared to juveniles to higher CO_2_ may be partly related to the types of CaCO_3_ each stage synthesizes. The first CaCO_3_ secreted by bivalve larvae is likely amphorous calcium carbonate, a precursor to aragonite or calcite that is 50-fold more susceptible to carbonate dissolution compared to the forms of CaCO_3_ (aragonite, calcite) primarily found in juvenile stage bivalves [81]. Regarding *A. irradians*, juveniles and particularly larvae were highly sensitive to elevated temperature and somewhat less affected by CO_2_. As such, the future success of this species may be highly dependent on the ability of all developmental stages to cope with temperature stress.

Many coastal ecosystems already experience elevated levels of CO_2_ [5], [6], [24], in part due to decomposition of naturally and anthropogenically derived organic matter [7], [8], [9], [10]. As these systems experience warming in the coming decades, a positive feedback loop may be established whereby increasing temperatures increase microbial remineralization rates of organic matter leading to further increases in CO_2_ concentrations. As such, further studies that concurrently examine the effects of increasing temperatures and CO_2_ concentrations on calcifying organisms in coastal marine ecosystems are certainly warranted.

Experimental research that seeks to mimic natural phenomena is inherently prone to limitations and this study was not an exception. For example, our delivery of a static level of CO_2_ and temperature during experiments may have elicited a more extreme response than those displayed by individuals in coastal ecosystems where natural variations in temperature and CO_2_ concentration may provide periods of stress and recovery that might permit some physiological compensation. In addition, like many other studies (e.g. [18], [19], [20], [21], [78]), our experiments with juvenile bivalves introduced animals reared under ideal conditions into experimental treatments, a procedure that does not mimic future ocean acidification but may be characteristic of some present day, coastal ocean acidification. In contrast, since the internal pH of adult bivalves is osmotically regulated and relatively static, developing gametes persist under ideal chemical conditions until spawned [29], [30], [31], [34]. Once spawned, larvae suddenly enter a new chemical environment that differs from the biochemical stability offered by their parent. Similarly our experiments introduced bivalve larvae into experimental vessels within hours of fertilization. As coastal oceans acidify over the next two centuries, there may be selection pressure on bivalves to become more resistant to high CO_2_ but it seems less likely that selection will alter homeostatic processes that regulate internal pH of adult bivalves [34]. As such, in the future, bivalve larvae may experience elevated CO_2_ in a manner similar to our experiment design: Persisting under ideal conditions as gametes and then thrust into a new, high temperature or CO_2_ environment as larvae. Therefore, while some aspects of this research had limitations, introducing hours-old larvae into a new environment may be one of the more realistic experimental approaches attempting to mimic future ocean acidification.

The sum of environmental stressors that may affect marine organisms in the coming decades, particularly in coastal ecosystems, is substantial. Exactly how increased temperature and CO_2_ concentrations will combine to affect bivalve populations is still not entirely understood. This study demonstrates the negative consequences of developing in a thermally stressed and acidified environment for larval and juvenile bivalves. These effects may have serious implications for the future of these bivalves and other marine calcifying organisms faced with global climate change.

## Supporting Information

Figure S1 **Percent metamorphosed of** ***M. mercenaria*** **larvae grown under three levels of CO_2_, approximately 250, 390, and 750 ppm, and two temperatures 24°C and 28°C (** **Table 1** **).** Error bars represent standard deviation of replicated vessels per treatment (*n* = 4 per treatment).
(TIF)

Figure S2 **Percent metamorphosed of** ***A.irradians*** **larvae grown under three levels of CO_2_, approximately 250, 390, and 750 ppm, and two temperatures 24°C and 28°C (** **Table 1** **).** Error bars represent standard deviation of replicated vessels per treatment (*n* = 4 per treatment).
(TIF)

Table S1 **Two-way analysis of variance tables for all experiments.**
(DOC)
