# Supplementary figures and images for: Effects of Elevated Temperature and Carbon Dioxide on the Growth and Survival of Larvae and Juveniles of Three Species of Northwest Atlantic Bivalves
Source: PLoS One. 2011 Oct 31;6(10):e26941. doi: 10.1371/journal.pone.0026941 (PMC3204984; doi:10.1371/journal.pone.0026941)

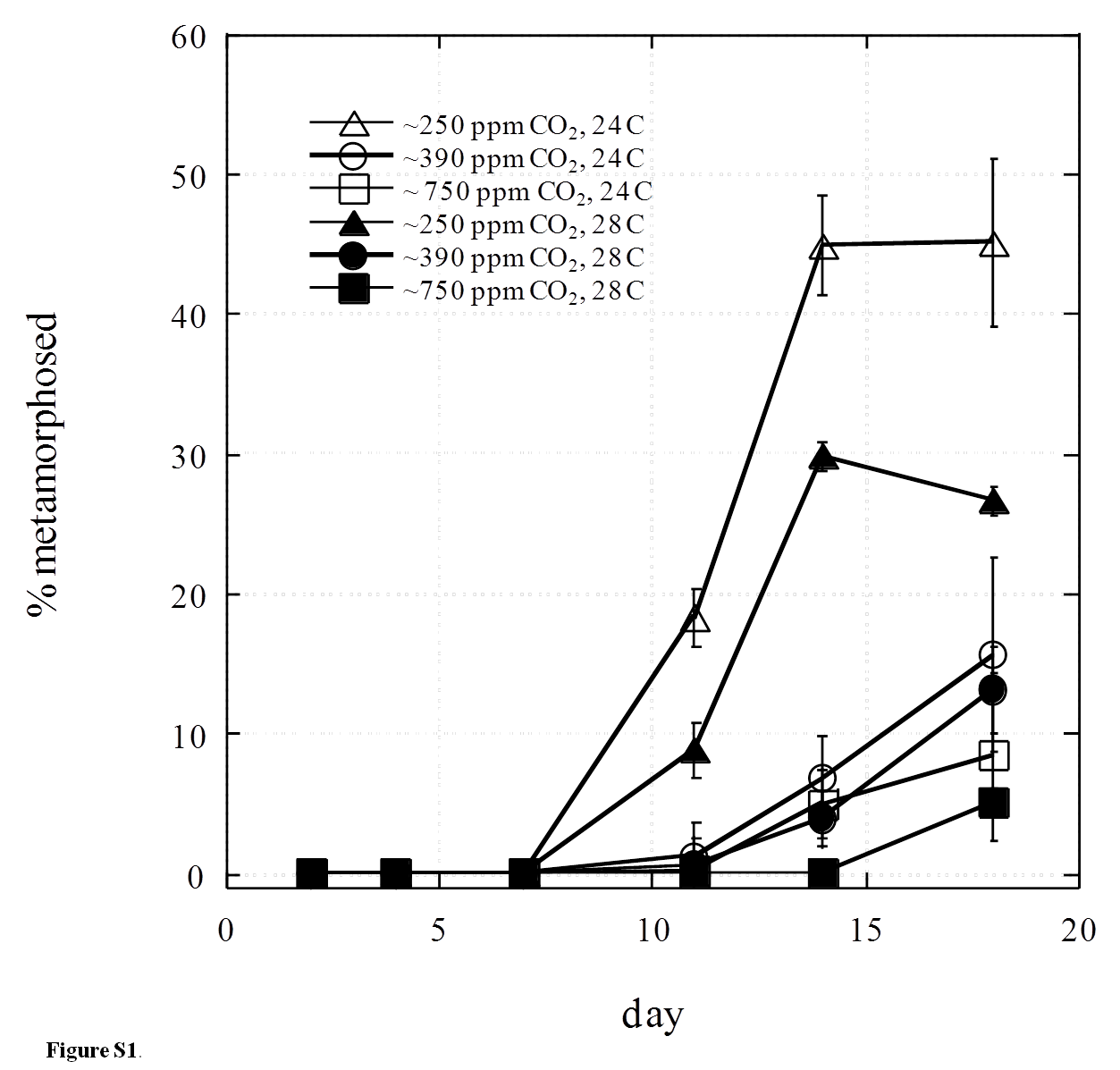

Supplement: Figure S1 — Percent metamorphosed of M. mercenaria larvae grown under three levels of CO2, approximately 250, 390, and 750 ppm, and two temperatures 24°C and 28°C ( Table 1 ). Error bars represent standard deviation of replicated vessels per treatment (n = 4 per treatment). (TIF) [file pone.0026941.s001.tif]

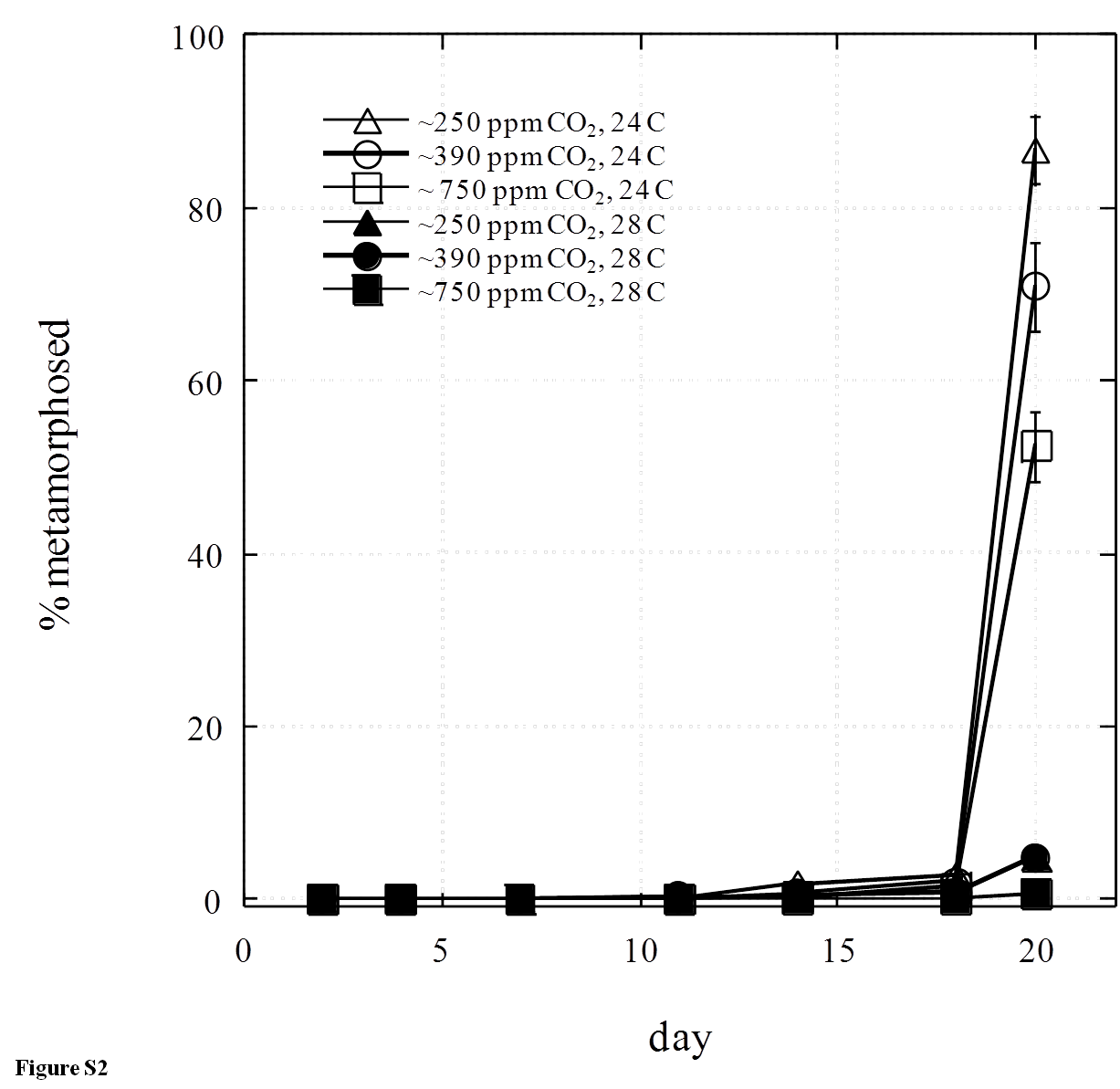

Supplement: Figure S2 — Percent metamorphosed of A.irradians larvae grown under three levels of CO2, approximately 250, 390, and 750 ppm, and two temperatures 24°C and 28°C ( Table 1 ). Error bars represent standard deviation of replicated vessels per treatment (n = 4 per treatment). (TIF) [file pone.0026941.s002.tif]
