# Supplementary material for: Effects of Elevated Temperature and Carbon Dioxide on the Growth and Survival of Larvae and Juveniles of Three Species of Northwest Atlantic Bivalves
Source: PLoS One. 2011 Oct 31;6(10):e26941. doi: 10.1371/journal.pone.0026941 (PMC3204984; doi:10.1371/journal.pone.0026941)
Supplement: Table S1 — Two-way analysis of variance tables for all experiments. (DOC) [file pone.0026941.s003.doc]

**Two-way analysis of variance for *Mercenaria mercenaria* larvae metamorphosis.**

| Source of variation | df | SS | MS | F-ratio | *p-value* |
| --- | --- | --- | --- | --- | --- |
| CO_2_ level | 2 | 3,641.928 | 1,820.964 | 277.661 | <0.001 |
| Temperature level | 1 | 394.071 | 394.071 | 60.088 | <0.001 |
| CO_2_ * temperature | 2 | 327.774 | 163.887 | 24.990 | <0.001 |
| Error | 18 | 118.048 | 6.558 |  |  |

**Two-way analysis of variance for *Mercenaria mercenaria* larvae survival.**

| Source of variation | df | SS | MS | F-ratio | *p-value* |
| --- | --- | --- | --- | --- | --- |
| CO_2_ level | 2 | 1,335.393 | 667.696 | 232.680 | <0.001 |
| Temperature level | 1 | 1,800.600 | 1,800.600 | 627.477 | <0.001 |
| CO_2_ * temperature | 2 | 168.158 | 84.079 | 29.300 | <0.001 |
| Error | 18 | 51.653 | 2.870 |  |  |

**Two-way analysis of variance for *Mercenaria mercenaria* larvae size.**

| Source of variation | df | SS | MS | F-ratio | *p-value* |
| --- | --- | --- | --- | --- | --- |
| CO_2_ level | 2 | 158,867.802 | 79,433.901 | 703.999 | <0.001 |
| Temperature level | 1 | 2,784.260 | 2,784.260 | 24.676 | <0.001 |
| CO_2_ * temperature | 2 | 725.271 | 362.635 | 3.214 | 0.064 |
| Error | 18 | 2,030.983 | 112.832 |  |  |

**Two-way analysis of variance for *Mercenaria mercenaria* larvae lipid synthesis.**

| Source of variation | df | SS | MS | F-ratio | *p-value* |
| --- | --- | --- | --- | --- | --- |
| CO_2_ level | 2 | 0.109 | 0.055 | 40.386 | <0.001 |
| Temperature level | 1 | 0.036 | 0.036 | 26.845 | <0.001 |
| CO_2_ * temperature | 2 | 0.005 | 0.002 | 1.702 | 0.210 |
| Error | 18 | 0.024 | 0.001 |  |  |

**Two-way analysis of variance for *Argopecten irradians* larvae metamorphosis.**

| Source of variation | df | SS | MS | F-ratio | *p-value* |
| --- | --- | --- | --- | --- | --- |
| CO_2_ level | 2 | 1,486.349 | 743.174 | 609.495 | <0.001 |
| Temperature level | 1 | 26,814.716 | 26,814.716 | 21,991.384 | <0.001 |
| CO_2_ * temperature | 2 | 904.764 | 452.382 | 371.009 | <0.001 |
| Error | 18 | 21.948 | 1.219 |  |  |

**Two-way analysis of variance for *Argopecten irradians* larvae survival.**

| Source of variation | df | SS | MS | F-ratio | *p-value* |
| --- | --- | --- | --- | --- | --- |
| CO_2_ level | 2 | 2,984.929 | 1,492.464 | 1,004.938 | <0.001 |
| Temperature level | 1 | 8,434.412 | 8,434.412 | 5,679.239 | <0.001 |
| CO_2_ * temperature | 2 | 385.544 | 192.772 | 129.801 | <0.001 |
| Error | 18 | 26.732 | 1.485 |  |  |

**Two-way analysis of variance for *Argopecten irradians* larvae size.**

| Source of variation | df | SS | MS | F-ratio | *p-value* |
| --- | --- | --- | --- | --- | --- |
| CO_2_ level | 2 | 159,159.198 | 79,579.599 | 1,120.054 | <0.001 |
| Temperature level | 1 | 4,054.940 | 4,054.940 | 57.072 | <0.001 |
| CO_2_ * temperature | 2 | 396.660 | 198.330 | 2.791 | 0.088 |
| Error | 18 | 1,278.896 | 71.050 |  |  |

**Two-way analysis of variance for *Argopecten irradians* larvae lipid synthesis.**

| Source of variation | df | SS | MS | F-ratio | *p-value* |
| --- | --- | --- | --- | --- | --- |
| CO_2_ level | 2 | 0.071 | 0.036 | 74.069 | <0.001 |
| Temperature level | 1 | 0.002 | 0.002 | 4.468 | 0.049 |
| CO_2_ * temperature | 2 | 0.000 | 0.000 | 0.402 | 0.675 |
| Error | 18 | 0.009 | 0.000 |  |  |

**Two-way analysis of variance for *Mercenaria mercenaria* juvenile shell growth.**

| Source of variation | df | SS | MS | F-ratio | *p-value* |
| --- | --- | --- | --- | --- | --- |
| CO_2_ level | 1 | 941.994 | 941.994 | 0.489 | 0.487 |
| Temperature level | 1 | 14,955.545 | 14,955.545 | 7.762 | 0.007 |
| CO_2_ * temperature | 1 | 5,966.111 | 5,966.111 | 3.096 | 0.083 |
| Error | 116 |  |  |  |  |

**Two-way analysis of variance for *Mercenaria mercenaria* juvenile tissue growth.**

| Source of variation | df | SS | MS | F-ratio | *p-value* |
| --- | --- | --- | --- | --- | --- |
| CO_2_ level | 1 | 0.000 | 0.000 | 0.000 | 0.995 |
| Temperature level | 1 | 0.001 | 0.001 | 0.514 | 0.475 |
| CO_2_ * temperature | 1 | 0.000 | 0.000 | 0.000 | 0.995 |
| Error | 116 | 0.319 | 0.003 |  |  |

**Two-way analysis of variance for *Crassostrea virginica*  juvenile shell growth.**

| Source of variation | df | SS | MS | F-ratio | *p-value* |
| --- | --- | --- | --- | --- | --- |
| CO_2_ level | 1 | 167,123.761 | 167,123.761 | 4.279 | 0.044 |
| Temperature level | 1 | 12,595.675 | 12,595.675 | 0.322 | 0.573 |
| CO_2_ * temperature | 1 | 2.714 | 2.714 | 0.000 | 0.993 |
| Error | 116 | 1,992,072.311 | 39,060.241 |  |  |

**Two-way analysis of variance for *Crassostrea virginica* juvenile tissue growth.**

| Source of variation | df | SS | MS | F-ratio | *p-value* |
| --- | --- | --- | --- | --- | --- |
| CO_2_ level | 1 | 0.001 | 0.001 | 0.034 | 0.854 |
| Temperature level | 1 | 0.004 | 0.004 | 0.114 | 0.736 |
| CO_2_ * temperature | 1 | 0.000 | 0.000 | 0.006 | 0.938 |
| Error | 116 | 4.241 | 0.037 |  |  |

**Two-way analysis of variance for *Argopecten irradians* juvenile shell growth**.

| Source of variation | df | SS | MS | F-ratio | *p-value* |
| --- | --- | --- | --- | --- | --- |
| CO_2_ level | 1 | 82.214 | 82.214 | 0.007 | 0.933 |
| Temperature level | 1 | 84,312.768 | 84,312.768 | 7.347 | 0.008 |
| CO_2_ * temperature | 1 | 2,515.215 | 2,515.215 | 0.219 | 0.641 |
| Error | 116 | 1,009,898.506 | 11,476.119 |  |  |

**Two-way analysis of variance for *Argopecten irradians*** juvenile tissue growth.

| Source of variation | df | SS | MS | F-ratio | *p-value* |
| --- | --- | --- | --- | --- | --- |
| CO_2_ level | 1 | 0.001 | 0.001 | 0.167 | 0.693 |
| Temperature level | 1 | 0.035 | 0.035 | 9.362 | 0.016 |
| CO_2_ * temperature | 1 | 0.000 | 0.000 | 0.047 | 0.834 |
| Error | 117 | 0.030 | 0.004 |  |  |

**Two-way analysis of variance for *Argopecten irradians* juvenile survival.**

| Source of variation | df | SS | MS | F-ratio | *p-value* |
| --- | --- | --- | --- | --- | --- |
| CO_2_ level | 1 | 1,875.000 | 1,875.000 | 5.625 | 0.045 |
| Temperature level | 1 | 675.000 | 675.000 | 2.025 | 0.193 |
| CO_2_ * temperature | 1 | 75.000 | 75.000 | 0.225 | 0.648 |
| Error | 117 | 2,666.667 | 333.333 |  |  |

**Two-way analysis of variance for *Mercenaria mercenaria* juvenile survival.**

| Source of variation | df | SS | MS | F-ratio | *p-value* |
| --- | --- | --- | --- | --- | --- |
| CO_2_ level | 1 | 2.508 | 2.508 | 0.204 | 0.653 |
| Temperature level | 1 | 3.142 | 3.142 | 0.255 | 0.614 |
| CO_2_ * temperature | 1 | 2.508 | 2.508 | 0.204 | 0.653 |
| Error | 116 | 1,428.320 | 12.313 |  |  |

**Two-way analysis of variance for *Crassostrea virginica* juvenile survival.**

| Source of variation | df | SS | MS | F-ratio | *p-value* |
| --- | --- | --- | --- | --- | --- |
| CO_2_ level | 1 | 34.143 | 34.143 | 1.133 | 0.289 |
| Temperature level | 1 | 12.798 | 12.798 | 0.425 | 0.516 |
| CO_2_ * temperature | 1 | 2.125 | 2.125 | 0.070 | 0.791 |
| Error | 116 | 3,497.056 | 30.147 |  |  |
